# Assessment of a Silicon-Photomultiplier-Based Platform for the Measurement of Intracellular Calcium Dynamics with Targeted Aequorin

**DOI:** 10.1021/acssensors.0c00277

**Published:** 2020-07-23

**Authors:** Federico Alessandro Ruffinatti, Samuela Lomazzi, Luca Nardo, Romualdo Santoro, Alexander Martemiyanov, Marianna Dionisi, Laura Tapella, Armando A. Genazzani, Dmitry Lim, Carla Distasi, Massimo Caccia

**Affiliations:** † Department of Pharmaceutical Sciences, Università del Piemonte Orientale, Via Bovio 6, Novara 28100, Italy; ‡ Department of Science and High Technology, Università degli Studi dell’Insubria, Via Valleggio 11, Como 22100, Italy; § ITEP, Bol’shaya Cheremushkinskaya Ulitsa, 25, Moscow 117218, Russia

**Keywords:** silicon photomultipliers, aequorin, calcium signaling, live cell, bioluminescence

## Abstract

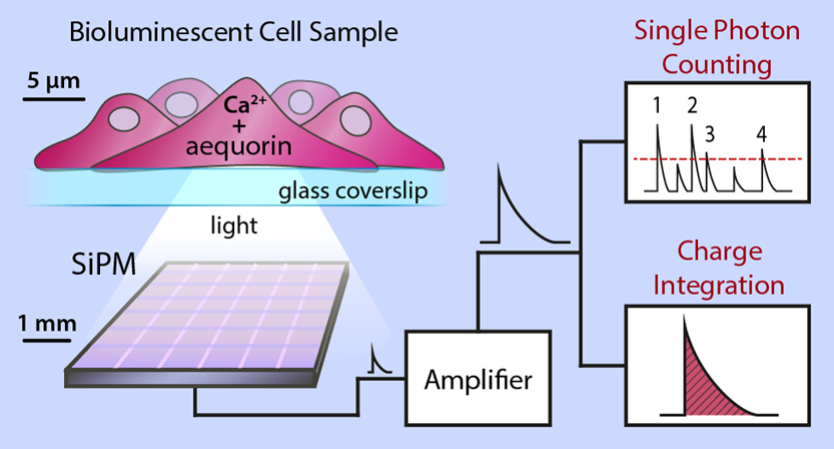

Ca^2+^ is among the most important intracellular second messengers participating in a plethora of biological processes, and the measurement of Ca^2+^ fluctuations is significant in the phenomenology of the underlying processes. Aequorin-based Ca^2+^ probes represent an invaluable tool for reliable measurement of Ca^2+^ concentrations and dynamics in different subcellular compartments. However, their use is limited due to the lack on the market of ready-to-use, cost-effective, and portable devices for the detection and readout of the low-intensity bioluminescence signal produced by these probes. Silicon photomultipliers (SiPMs) are rapidly evolving solid-state sensors for low light detection, with single photon sensitivity and photon number resolving capability, featuring low cost, low voltage, and compact format. Thus, they may represent the sensors of choice for the development of such devices and, more in general, of a new generation of multipurpose bioluminescence detectors suitable for cell biology studies. Ideally, a detector customized for these purposes must combine high dynamic range with high fidelity in reconstructing the light intensity signal temporal profile. In this article, the ability to perform aequorin-based intracellular Ca^2+^ measurements using a multipurpose, low-cost setup exploiting SiPMs as the sensors is demonstrated. SiPMs turn out to assure performances comparable to those exhibited by a custom-designed photomultiplier tube-based aequorinometer. Moreover, the flexibility of SiPM-based devices might pave the way toward routinely and wide scale application of innovative biophysical protocols.

Calcium (Ca^2+^) is known to play a pivotal role in cell metabolism and signaling pathways, both as one of the electrolytes responsible for membrane potential and as an ubiquitous intracellular second messenger, able to activate or modulate the activity of a plethora of proteins.^1^ Cells continuously work to extrude calcium from cytosol by both expelling it in the extracellular space and storing it within suitable intracellular subcompartments including endoplasmic reticulum, mitochondria, and lysosomes.^2^ As a consequence of these processes, cytosolic free calcium concentration ([Ca^2+^]_c_) is much lower than the typical extracellular values (50–100 nM compared to 1.5–2 mM), thus making Ca^2+^ signaling extremely effective and noiseless.^3^ Both calcium influx from extracellular space and/or release from internal stores can occur as a consequence of the binding of specific membrane receptors with their ligands.^4^ As a result, [Ca^2+^]_c_ can change following complex spatiotemporal dynamics, conveying information coded in amplitude, frequency, and temporal profile of the signal^5,6^ eventually mediating different functions in a cell-type-specific way. Accordingly, [Ca^2+^]_c_ is traditionally one of the most investigated biophysical parameters to understand cell physiology and pathology.

Techniques for Ca^2+^ sensing include fluorescence and bioluminescence assays.^7,8^ In particular, fluorescent synthetic Ca^2+^ probes like Fura-2 and Fluo-4^9^ have become popular due to their ease of use and the wide distribution of fluorescent microscopes. On the other hand, genetically encoded calcium indicators (GECIs) offer a reliable mean to detect and measure Ca^2+^ in specific subcellular locations. These probes include fluorescent probes (e.g., the GFP-derived GCaMP, and GeCO),^10−12^ fluorescence resonance energy transfer (FRET)-based Ca^2+^ probes (e.g., cameleons and their analogues),^13,14^ luminescent Ca^2+^ probes (e.g., AEQ-based indicators), and bioluminescence resonance energy transfer (BRET)-based Ca^2+^ indicators (the last two reviewed in ref (15)). Among these techniques, aequorin bioluminescence offers outstanding advantages.^8,16^ Aequorin (AEQ) is a 21 kDa bioluminescent protein that can be used as a calcium reporter without being externally excited. More in detail, it is an enzyme that, upon the binding of three Ca^2+^ ions, catalyzes the oxidation of its substrate coelenterazine, ultimately producing a blue light emission (peaked at 465 nm):
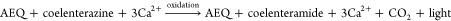


Many engineered aequorin variants are currently available on the market, and many different cell types can be transfected to make them express aequorin at cytosolic level rather than just in some specific subcellular organelles, such as mitochondria. In brief, aequorin represents a natural calcium reporter, targetable, highly biocompatible, that does not interfere with cell calcium homeostasis. Moreover, unlike fluorescence, bioluminescence does not require any excitation light, thus preserving cells from photodamage, while featuring a wide dynamic range in emission. Despite all these indisputable advantages, typical aequorin emission intensities are very low if compared to those of fluorescent probes. Thus, extremely sensitive detectors are needed. Overall, the lack of a ready-to-use commercial setup and the high cost of specific-purpose custom-made detection systems (aequorinometers) represent the main limitations to aequorin widespread use.

Up to now, photomultiplier tubes (PMTs) have been the detectors of choice in aequorinometers because of their sensitivity up to the single photon level. However, PMTs are bulky, relatively fragile, and require biasing voltages in excess of 1 kV. In this regard, in the last 20 years, a new generation of solid-state photodetectors, known as silicon photomultipliers (SiPMs), has become commercially available, and it is now increasingly being regarded as the most promising competitor of PMTs.^17−19^ SiPMs can produce a current pulse of several tens of nanoseconds duration, containing millions of electrons in response to the absorption of a single photon, thus providing a gain comparable to that of PMTs in combination with all the benefits of a solid-state sensor: single photon sensitivity, low-voltage operation, small size, insensitivity to magnetic fields, mechanical robustness, and scalability. Technology-wise, a SiPM is an array of p–n junctions (microcells) operated in reverse bias mode beyond the breakdown voltage.^20−23^ A SiPM can be operated in two modes: single photon counting (SPC) and charge integration (CI).^24^ SPC is more suitable when the signal intensity is so low that the incoming photons can be distinguished. Conversely, it is limited at high frequencies by the superposition of single photon signals (pile-up). The pile-up limit depends on the pulse duration of the SiPM output signal and may be improved by properly shaping and filtering the native signal. The pile-up probability increases exponentially as a function of the impinging photons frequency, limiting the maximum detectable frequency to a few megahertz. CI allows accessing the information related to the charge collected in a time interval. Charge integration obviously overcomes the problem of pile-up, showing sensibility also at high frequencies, above 50 MHz. In this article, the ability to perform aequorin-based intracellular Ca^2+^ gradient measurements and time-course recordings exploiting SiPMs as sensors is demonstrated using a multipurpose, low-cost setup in which the SPC and CI acquisition modes are implemented in parallel to achieve the desired dynamic range and sensitivity. Namely, the sensitivity, dynamic range, and linearity of the system response are qualified on a simplified specimen consisting in a lysate of aequorin-transfected cells. In addition, [Ca^2+^]_c_ is monitored over time in AEQ-transfected HeLa cell cultures stimulated by the paradigmatic agonist ATP by exploiting both acquisition modalities.

## Instrumentation, Materials, and Methods

### SiPM Sensor and Front-End Electronics

The measurements presented in this work were performed using a HAMAMATSU S13360-6050CS (https://www.hamamatsu.com/resources/pdf/ssd/s13360_series_kapd1052e.pdf) sensor, integrating 14,400 cells with 50 μm pitch, resulting in a total area of 6 × 6 mm^2^. The detector features a peak photon detection efficiency of 40% in the blue (i.e., aequorin emission band), and it is characterized by an output-pulse full-time development of 150 ns and dark-count rate (DCR) at the megahertz level at room temperature (RT). The sensor was biased through the CAEN SP5600 power supply and amplification unit (PSAU; https://www.caen.it/products/sp5600e/).

The SiPM output signal was amplified by a custom low-noise and inverting amplifier featuring an AC-coupled high gain branch (34 dB) and a DC-coupled low gain one (20 dB). This latter was exploited for the CI measurements, making the system sensitive to photon frequencies so high that the baseline restoration is not possible, while the high gain branch was used for the SPC modality. See the Supporting Information for the detailed specifics of the twin gain amplifier and a schematic of the circuit (Figure S1).

In order to enhance the SPC capability of the system, the 34 dB amplified signal was shortened by means of a zero-pole cancellation circuit, reaching a full-time development of 30 ns (see ref (25) and the Supporting Information for further details). This corresponded to a 5% probability of pile-up events at 2 MHz count rate assuming a Poissonian temporal distribution of impinging photons. Then, the signal was discriminated by a leading-edge comparator and pulses were counted in 100 ms windows by a 16-bit scaler. Both the comparator and the scaler are integrated within the PSAU. The leading-edge discriminator threshold was set to 50% of the peak amplitude for a signal generated by a single detected photon. The data were processed in real time exploiting the SiPM kit control proprietary software provided by CAEN.

For CI, the low gain branch of the amplifier was fed into an analog integrator, namely, a CAEN V752N (https://www.caen.it/products/v792n/) charge-to-digital converter (QDC). The amplification factor and the integration time were tuned (in the ranges 2.5x–10x and 4 μs–32 μs, respectively) to cope with the QDC dynamic range. The sampling rate of the acquisition system was 11.2 kHz. Charge values were averaged over 5600 samples to increase sensitivity and yield intensity vs time traces with the same time granularity as those obtained by SPC.

**Figure 1:**
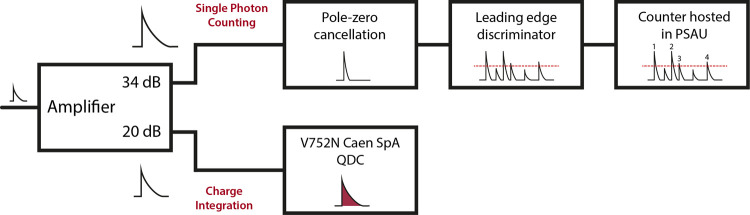
Block diagram of the SiPM-based experimental setup. The SiPM signal is amplified by a two-branch, custom-made amplifier. On one hand, the high gain branch (34 dB) is exploited for the SPC measurements. Signal length is shortened through a pole-zero cancellation circuit, and then each single photon pulse is counted through a leading-edge discriminator and a counter hosted on the PSAU (power supply and amplifier unit SP5600E by Caen S.p.A.). On the other hand, the low gain branch (20 dB) is used to perform the CI measurements. The signal is directly fed into a V752N analog integrator (Caen S.p.A.).

A block diagram summarizing the measurement setup is depicted in Figure 1.

### Sample to SiPM Interfacing

The SiPM sensor was mounted on a dedicated holder featuring an external diameter of 5 cm. A steel watertight chamber consisting of a base ring and a threaded insert was used to mount the coverslip hosting the bioluminescent samples (cell culture or cell lysate containing aequorin). Such a chamber was then equipped with an injection system consisting of a flexible tube to administer the chemicals of interest (i.e., Ca^2+^, ATP, and Triton X-100) and finally placed over the SiPM holder. A black cap fitting the outer diameter of the SiPM holder was used for covering both the sensor and the sample in order to protect them from environmental light. All measurements were performed at RT, while the SiPM temperature was constantly monitored through a thermistor placed in contact with the external packaging of the sensor. The thermal excursion observed during each single experiment was always within 0.5 °C.

### Experimental Determination of Sensitivity in Charge-Integration Modality

To assess the minimum photoelectron rate detectable in CI, a dedicated setup was devised. Illumination by a train of stochastically impinging single-photon pulses, which best approaches the condition occurring in the calcium sensing experiments, was mimicked as follows. The SiPM was illuminated at stochastic time intervals by the light pulses generated by a PLS-8-2-443 subnanosecond-pulsed LED (PicoQuant), driven by the PDL 800-B driver (PicoQuant) used in the external trigger mode. Stochastic triggers at different frequencies were obtained setting a tunable threshold on a white-noise trace generated using a built-in function in an Agilent 33250A waveform generator. The (Poisson) photon number distribution of the LED pulses was measured at each threshold, and the intensity was adjusted in order to have an average value of 1 photon per pulse. The amplified detector output signal was directly fed into the QDC and integrated over 5 μs.

### Cell Cultures and Infection

Human cervical adenocarcinoma cell line (HeLa, ATCC CCL-2) was cultured in complete DMEM medium (Dulbecco’s modified Eagle’s medium, Sigma, Cat. D5671) supplemented with 10% fetal bovine serum (FBS, Gibco, Cat. 10270), 2 mM glutamine (Sigma, Cat. G7513), 100 U/mL penicillin, and 0.1 mg/mL streptomycin (Sigma, Cat. P0781). HeLa cells stably expressing cytosol-targeted aequorin (cyt-RFP-AEQ) were generated by infecting 50,000 cells with lentiviral particles at a multiplicity of infection (MOI) of 4–6. After three passages, the cells expressing medium-high levels of cyt-RFP-AEQ were enriched by fluorescence-activated cell sorting (FACS). After amplification, the cyt-RFP-AEQ-expressing HeLa (HeLa-cyt-AEQ) were stored in −80 °C until needed. Generation of lentiviral cyt-RFP-AEQ construct and production of lentiviral particles and virus titer definition were described elsewhere.^16^

### Calcium Measurements on Cell Lysates

For cell lysate preparation, HeLa-cyt-AEQ cells were grown in 100 mm culture dishes (Falcon, Cat. 353003). At confluence, cells were washed twice in PBS and scraped in 250 μL of a buffer containing (in mM): 150 Tris, 0.8 phenylmethylsulfonyl fluoride (PMSF), and 0.1 ethylenediaminetetraacetic acid (EDTA), pH 7.2. After three cycles of freeze-thawing, cells were centrifuged (12,000*g*, 5 min at 4 °C), the pellet was discarded while the supernatant was aliquoted and stored at −80 °C.

For AEQ reconstitution, 100 μL aliquots of HeLa-cyt-AEQ lysate were supplemented with 140 mM β-mercaptoethanol (Sigma, Cat. M6250) and 5 μM native coelenterazine (GoldBio, St. Luis, MO, Cat. CZ5) and allowed to reconstitute O.N. (15–24 h) on ice. For Ca^2+^ measurements, serial dilutions were prepared in 150 mM Tris, supplemented with 10 mM EDTA, pH 7.2.

For calcium measurements, 500 μL of cell lysate was aliquoted into the acquisition chamber. The latter was mounted on the top of SiPM using optical grease to assure optimal matching of the refractive index. After 30 s of acquisition, 1 mL of 150 mM Tris supplemented with 50 mM CaCl_2_ was injected in the acquisition chamber. After AEQ discharge, the trace was recorded until return to baseline (about 4 min).

### Calcium Measurements on Living Cultured Cells

For Ca^2+^ measurements in intact cells, HeLa-cyt-AEQ expressing cells were plated at decreasing densities (from 400,000 to 700 cells/well) on 24 mm round glass coverslips in 6-well plates 24 h before the experiment. In the day of measurements, cells were reconstituted with native coelenterazine by adding the prosthetic group directly to the culture medium at 5 μM for 1–3 h. Coverslips with the reconstituted cells were mounted in the acquisition chamber and placed on the top of SiPM using optical grease. After 30 s of acquisition, an increase in intracellular Ca^2+^ concentration was stimulated by injection of ATP (100 μM final concentration). Three to four minutes after ATP injection, cells were disrupted by administration of 0.1% Triton X-100 in water and residual active AEQ was discharged with 50 mM CaCl_2_. The acquisition continued until the trace returned to baseline.

## Results

### Response Linearity Assessment by Cell Lysates

The operational dynamic range of the system was qualified in terms of the response linearity using AEQ from cell lysate in order to exclude all those possible sources of biological variability that are intrinsic to cell culture samples. AEQ concentration was progressively diluted according to a geometric progression of common ratio *r* = 2. For each concentration, the same volume (500 μL) of sample was used.

**Figure 2:**
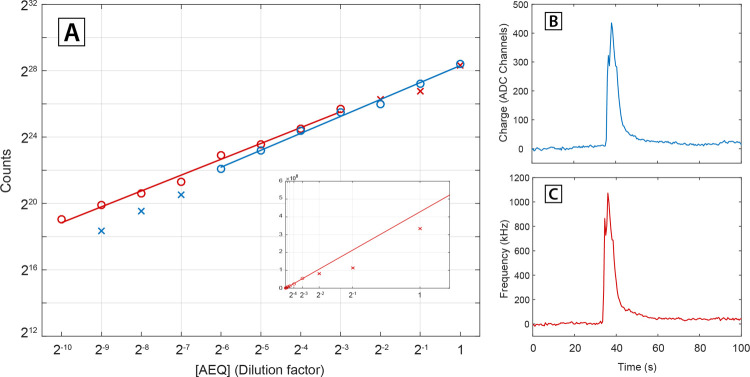
Operational dynamic range of the system qualified in terms of response linearity. (A) The integrated intensity of the Ca^2+^ signal (circles and crosses) is represented for both the acquisition modes (CI and SPC) as a function of AEQ dilution factor. CI data are in blue, while SPC data are in red. The inset shows SPC data points in the linear scale to emphasize the deviation from linearity due to pile-up phenomenon. In all the cases, crosses represent the data excluded from the linear fit. (B) An example of trace recorded by CI is shown for a dilution factor corresponding to 2^–5^. (C) The same signal shown in (B), as seen from the SPC branch. Notice how, at least in the superimposition region (from 2^–6^ to 2^–3^), the temporal profile of the signal is faithfully reproduced by both the modalities.

Bioluminescence was measured in parallel with the two techniques: SPC and CI. Regardless of the acquisition mode, traces were subjected to baseline subtraction and the integrated luminescence intensity (which is proportional to the total number of coelenterazine oxidation reactions, thus to aequorin concentration) was computed. In Figure 2, logarithmic plots of integrated luminescence intensity versus concentration are reported for CI (top) and SPC (bottom), respectively. The integrated charge values have been converted into their corresponding values expressed in photon counts by applying the conversion factor provided by datasheets (i.e., 272 fC/photoelectron^1^) in order to allow direct comparison between the two panels. For both acquisition modalities, the representative trace acquired at a dilution factor of 2^–5^ is also shown. It is also worth mentioning that, at the lowest tested concentration value, a detectable signal was observed only on the SPC branch.

Because in the log–log scale a function of the type *y* = *mx^a^* maps into a straight line with the slope equal to *a* and intercept equal to *q* = log (*m*), qualitative inspection of the plots suggests that the response of the system is roughly linear in both acquisition modalities over a concentration range spanning more than three orders of magnitude (log_10_2^11^ ≈ 3.31). Fitting the two plots to a linear model function yields quantitative information on deviations from response linearity as well as on sensitivity. Including all the data points in the fits results in the slope values *a*_CI_ = 1.107 ± 0.030 and *a*_SPC_ = 0.921 ± 0.027, indicating that both acquisition modes are plagued by slight deviations. Indeed, in the CI plot, the count value for the three points at lowest concentrations is sizably underestimated compared to the corresponding values determined in the SPC branch. This might be ascribed to insufficient sensitivity of the CI mode in this concentration range. Excluding these points from the linear regression results in a slope value *a*_CI_ = 1.02 ± 0.04, compatible with data linearity. Conversely, the slope obtained by fitting the whole SPC dataset indicates a sublinear increase in counts with increasing AEQ concentration, being the symptom of a phenomenon leading to saturation. The latter is apparent in the linear scale, as shown in the inset of Figure 2A. Excluding the three points at highest concentrations from linear regression yields a slope value compatible with data linearity, *a*_SPC_ = 0.95 ± 0.05. As mentioned above, the intercepts obtained by linear fit of the log–log plots quantify the sensitivity of the two acquisition modes. Their values are *q*_CI_ = 17.09 ± 0.34 and *q*_SPC_ = 17.90 ± 0.20, respectively, for the fitting runs with three excluded points, indicating comparable performances of the two modalities in this respect, though with a slight superiority of SPC. Namely, the quantity 2*^Δq^* = 1.75, where Δ*q* = *q*_SPC_ – *q*_CI_ represents the ratio between the sensitivities of the SPC and CI modes. In particular, this means that SPC is sensitive to a minimum concentration roughly 2-fold lower than CI, in agreement with experiments (see the point at [AEQ] = 2^–10^ in Figure 2A, which is detectable only in the SPC mode).

**Figure 3:**
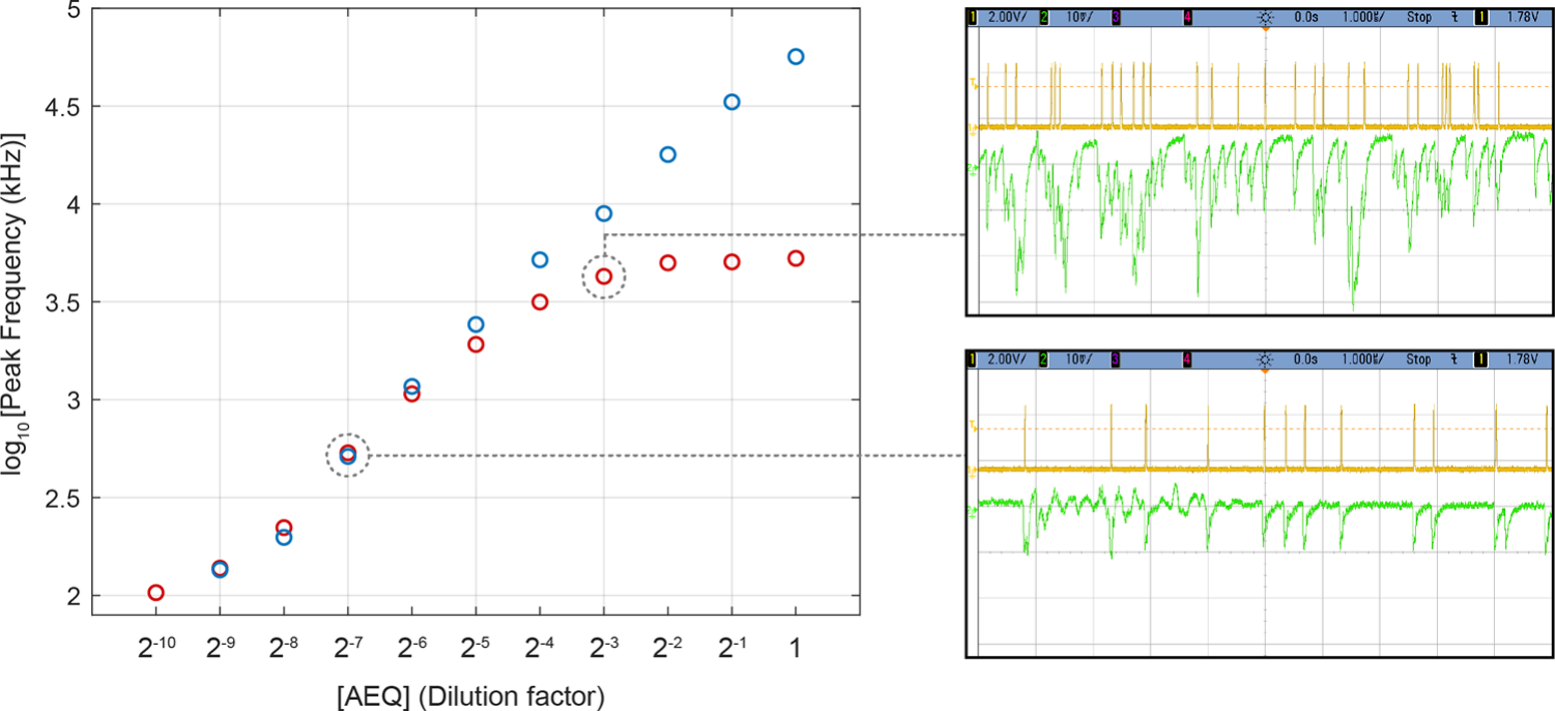
Assessment of the temporal-profile reconstruction capabilities. The Ca^2+^ signal peak frequency values detected in SPC (red) and CI (blue) are reported as a function of AEQ concentration in the log–log plot. On the right, two oscilloscope screenshots at two different concentrations show how the highest concentrations are affected by pile-up. In the screenshots, the green signal is referred to the SiPM output, while the yellow one is the output of the discriminator, which provides the information for the SPC mode.

Once the ability of the system to quantify the total amount of AEQ discharged during a calcium gradient event was assessed, the accuracy in reproducing the gradient temporal profile was taken into account. The first figure of merit evaluated to this aim was the reliability in retrieving the signal peak value. In the logarithmic plot of Figure 3, the peak frequency values detected in the SPC (red) and CI (blue) branches are reported as a function of concentration. The tendency of SPC data to saturate at high concentrations is much more evident than in the integral intensity plot. Inspection of oscilloscope screenshots of the pulse trains at the amplifier output shows a significant pile-up above ≈3 MHz (see exemplary traces reproduced in Figure 3). This phenomenon also contributes to the deviations from linearity observed in the plots of the Ca^2+^ signal-integrated intensity, although in the latter instance, it is mitigated by the fact that only the few time points around the peak are significantly affected (see Supporting Information, Figure S4 for a comparison of the traces acquired in CI and SPC branches in this concentration range). Moreover, the CI mode successfully determines the peak values even in the concentration range in which it was unable to assess accurate values of the integrated intensity (i.e., for the three lowest-concentration data points). This stems in support of the argument that the pitfalls of CI are connected to ultimate sensitivity (limit of detection, LoD) lower than SPC. In any case, the correct assessment of the peak values does not correspond to faithful reproduction of the Ca^2+^ signal temporal profile, as made apparent by direct comparison of the traces in the CI and SPC branches in this concentration range (see Supporting Informatio, Figure S5). More details about the pile-up effect and the limitations of both the modalities are reported in ref (25).

In conclusion, the simultaneous recording in SPC and CI modes highlights the advantages and disadvantages of the two approaches: while the QDC series show a reduced sensitivity at low concentrations essentially because of residual baseline fluctuations, counting modality results unsuitable for high aequorin concentrations due to the pile-up of the incoming pulses.

### Experimental Determination of the Limit of Detection in Photon-Counting and Charge-Integration Modes

**Figure 4:**
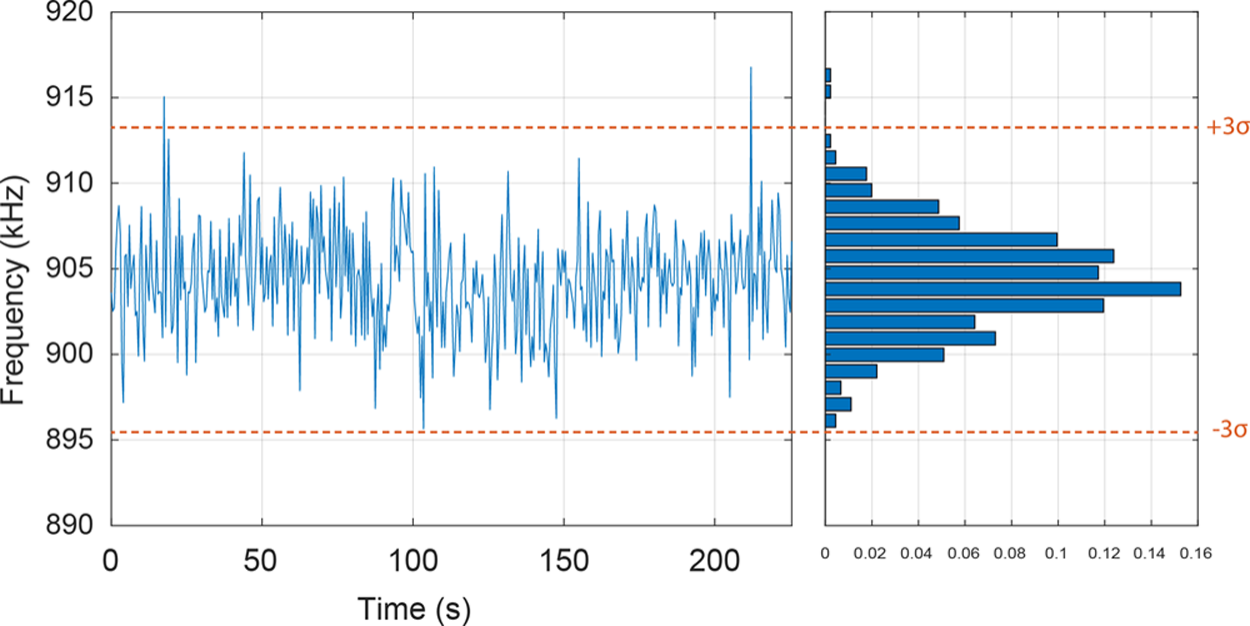
Dark-count rate (DCR) in the SPC mode. Left: an exemplary noise trace (blue line), recorded in the SPC mode. The two orange lines represent the experimental LoD limit that corresponds to ≈9 kHz. Right: the pertaining frequency histogram, showing a DCR level of (904 ± 3) kHz.

Since the LoD is a crucial parameter in the assessment of the instrument, its value was experimentally measured for both acquisition modes. In the SPC mode, the minimum detectable rate can be estimated by assuming the noise being solely due to DCR and obeying a Poisson statistics. For our detector kept in the dark, the average DCR was measured to be (904 ± 3) kHz. A representative DCR trace is shown in Figure 4, together with the pertaining frequency histogram. Defining the LoD as 3σ_DCR_, we get 9 kHz, which coincides with that theoretically expected for a Poisson distribution of noise fluctuations assuming a sampling time Δ*t* = 100 ms, as shown by the calculation below:
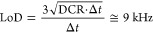
1

**Figure 5:**
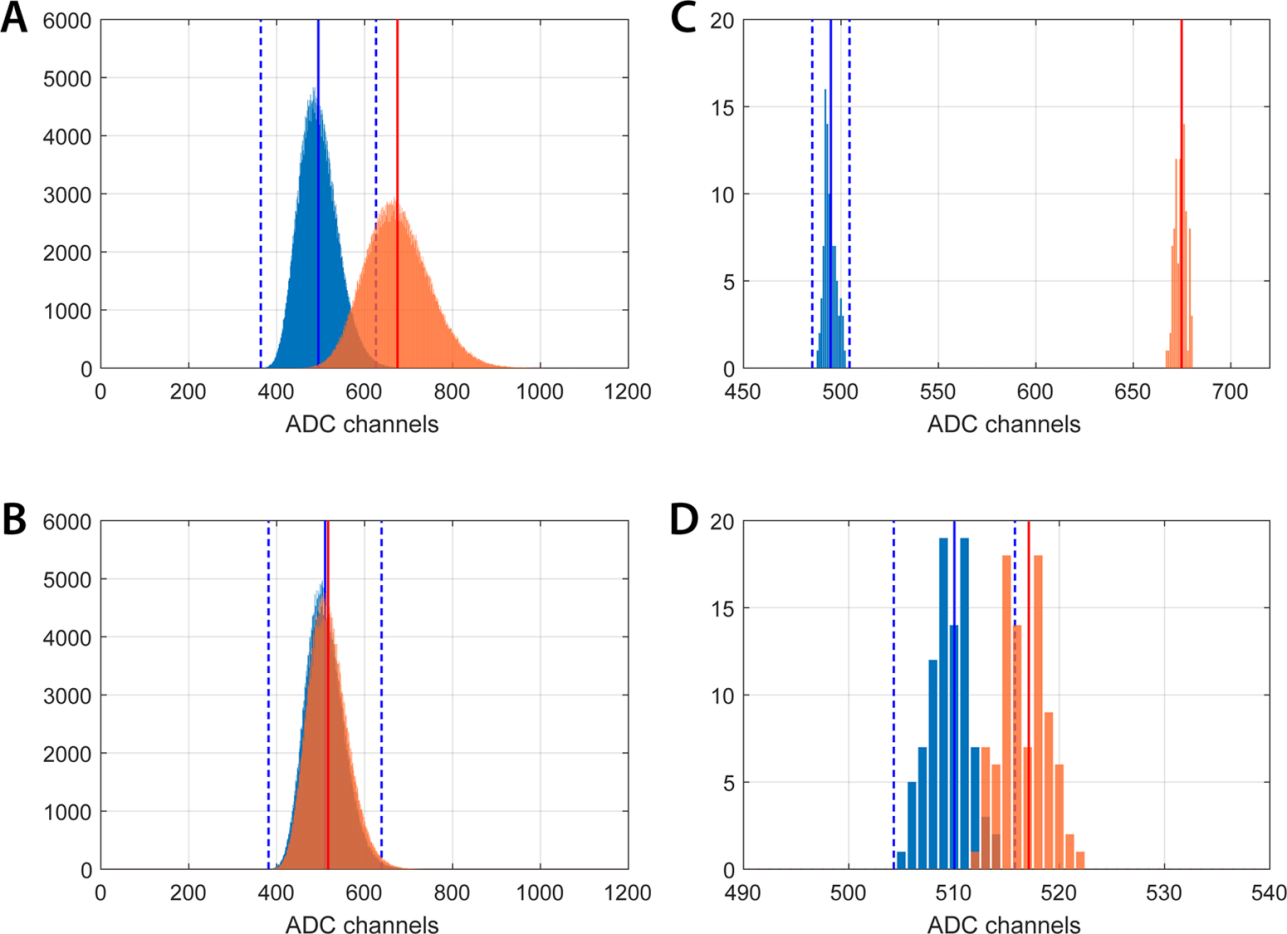
LoD assessment for the CI mode. (A) Charge distribution recorded in CI: in blue, the dark current of the biased SiPM (amplifier noise + SiPM DCR), the blue line is the mean value (μ), while the two dashed lines are the LoD reported as μ ± 3σ_DCR_ in an integration gate of 5 μs. In orange, the current due to the Poissonian light generated at a frequency of 2053 kHz is shown with the red line corresponding to its mean value. (B) Similarly, with a light frequency of 55 kHz. In (C) and (D), the current distributions after averaging over 5600 events are shown for 2053 and 55 kHz, respectively.

The experimentally measured DCR can also be exploited to estimate the theoretical LoD in the CI mode. For the integration gate Δ*t* = 5 μs used in the experiments, eq 1 yields an LoD value of 1.27 MHz. To quest this result, a dedicated measurement was performed. The dark current of the biased SiPM was measured within an integration gate of 5 μs. Subsequently, the SiPM was illuminated by a stochastic Poissonian light generated as detailed in Instrumentation, Materials, and Methods (see the Experimental Determination of Sensitivity in Charge-Integration Modality section). By increasing the pulse frequency, it was possible to assess the minimum frequency of incoming photons that could be discriminated from the noise. As shown in Figure 5A, the LoD resulted to be ≈2 MHz, possibly because of baseline fluctuations due to the amplifying stage. Setting a time granularity equal to the one used in the measurements performed on lysate (2 Hz) allowed averaging over a sample of 5600 consecutive events, thus reducing the LoD to ≈55 kHz (Figure 5B,D). It is worth mentioning that the improvement in LoD induced by averaging is lower than that expected assuming a Poisson distribution for the noise (i.e., ). Moreover, the LoD determined in the case of CI is higher than that measured for SPC by a factor 6.1, confirming that the photon-counting approach is more suitable for the detection of feeble signals. Finally, it should be taken into account that the SPC acquisition mode is in principle able to attain the same LoD as the CI mode by data sampling over a time ≈40-fold shorter, which might be crucial to reconstruct the temporal profile of very fast Ca^2+^ transients.

### Live-Cell Recording by QDC

**Figure 6:**
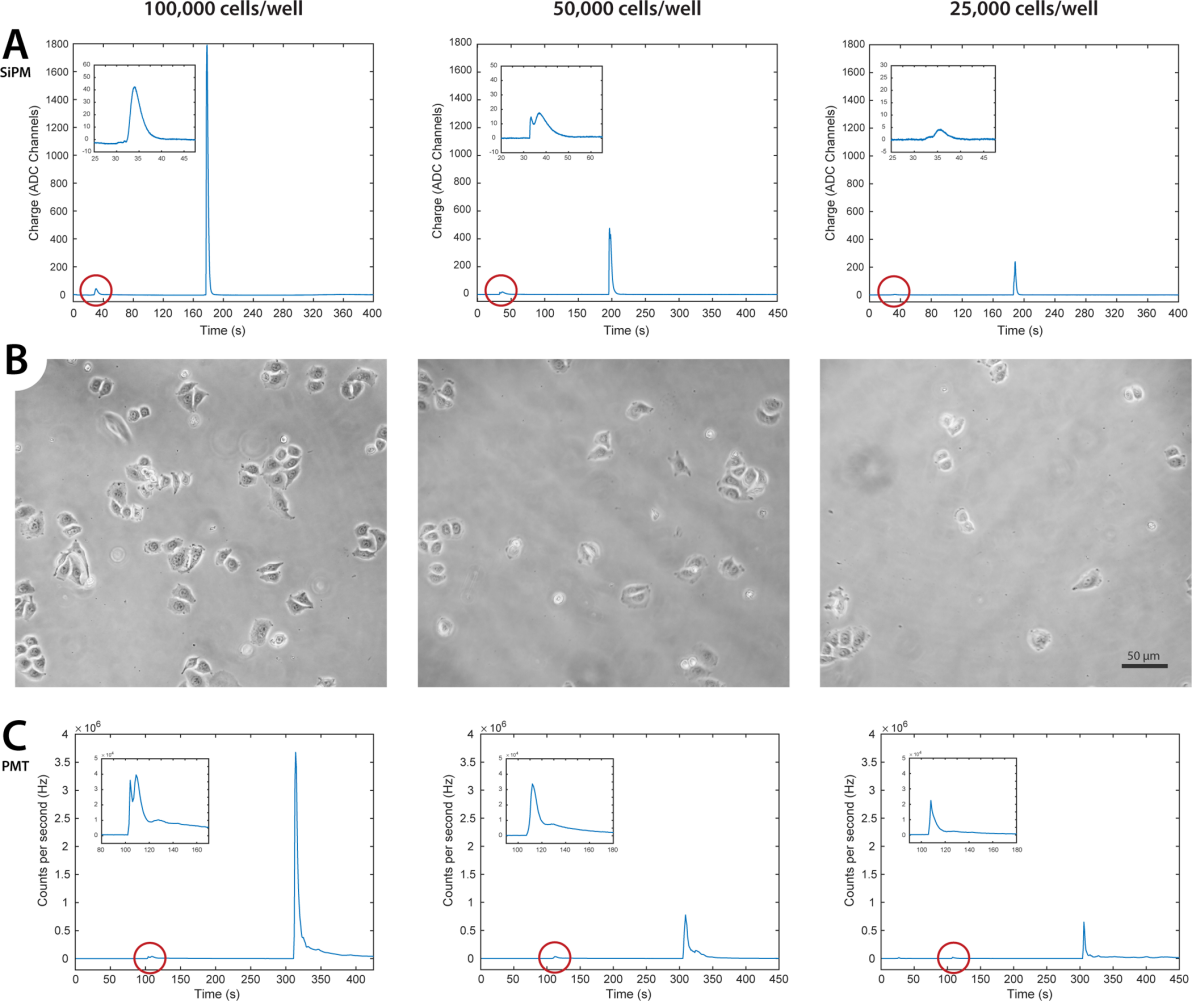
Bioluminescence signals from AEQ-transfected HeLa cells, recorded through QDC. (A) Three exemplificative traces of bioluminescence time courses recorded by CI and corresponding to plating densities of 100 × 10^3^, 50 × 10^3^, and 25 × 10^3^ cells/well respectively. For each trace, response to 100 μM ATP is circled in red and magnified in the upper-left inset. The higher subsequent peak represents the luminescent signal induced by Triton X-100. Notice the extreme dynamic range (from 5 to 1800 ADC channels) featured by the system. (B) Phase-contrast images of cultured HeLa cells used for bioluminescence recording. Each optical field is representative of the particular plating densities indicated above. (C) Three exemplificative traces of bioluminescence time courses as recorded by a PMT-based aequorinometer, corresponding to the same plating densities used in SiPM+QDC experiments (100 × 10^3^, 50 × 10^3^, and 25 × 10^3^ cells/well respectively). Even in this case, response to 100 μM ATP is circled in red and magnified in the upper-left inset, while the subsequent peak is the consequence of Triton X-100-induced membrane lysis.

A first series of experiments using intact HeLa cells was carried out recording bioluminescent signals from cell populations in the CI mode. A sampling time of 5 μs was used, and the traces were averaged over 1000 points. Cells were plated at different densities, from 100,000 to 25,000 cells/well (see Figure 6B). ATP, commonly used in calcium experiments for testing cell viability, was chosen as a prototypical agonist because of its capability to elicit a wide range of intracellular calcium signals, through both direct (e.g., purinergic) and indirect pathways, in an almost reproducible way. When injected, ATP induced a transient increase in luminescence, expression of the change in [Ca^2+^]_c_ (Figure 6A, red circles and insets). Normally, such a [Ca^2+^]_c_ increase was no more detectable after 1–2 min from ATP administration, in that the corresponding charge signal was again indistinguishable from the baseline value. After 3 min from the beginning of the experiment, 1 mL of Triton X-100 was injected into the chamber in order to destroy cell membranes and completely oxidize the total amount of coelenterazine by aequorin activity upon extracellular calcium binding. Experiments stopped after 7–8 min of total recording, a time long enough for capturing the entire signal elicited by Triton X-100 (Figure 6A). In order to assess the SiPM system performance, SiPM traces were compared to those recorded in similar experimental conditions by exploiting a custom-designed PMT-based aequorinometer produced by Cairn Research (UK), which is fully described in the Supporting Information (Figure S6). On a qualitative standpoint, the traces obtained by the two detection devices were comparable (see Figure 6C for exemplary aequorinometer traces). To obtain a quantitative comparison, the integral intensities of the ATP and Triton X-100 signals were separately evaluated after baseline subtraction and their average ratio *A*/*T* was computed as a figure of merit. Statistically compliant values, *A*/*T*_QDC_ = 0.039 ± 0.005 and *A*/*T*_PMT_ = 0.032 ± 0.009, were obtained for the SiPM- and the PMT-based systems, respectively. This feature indicates that the integrated charge by the SiPM provides an accurate recording of the light emitted in correspondence of membrane lysis at all the tested cell densities, without saturation onset.

### Live-Cell Recording in the Photon-Counting Mode

**Figure 7:**
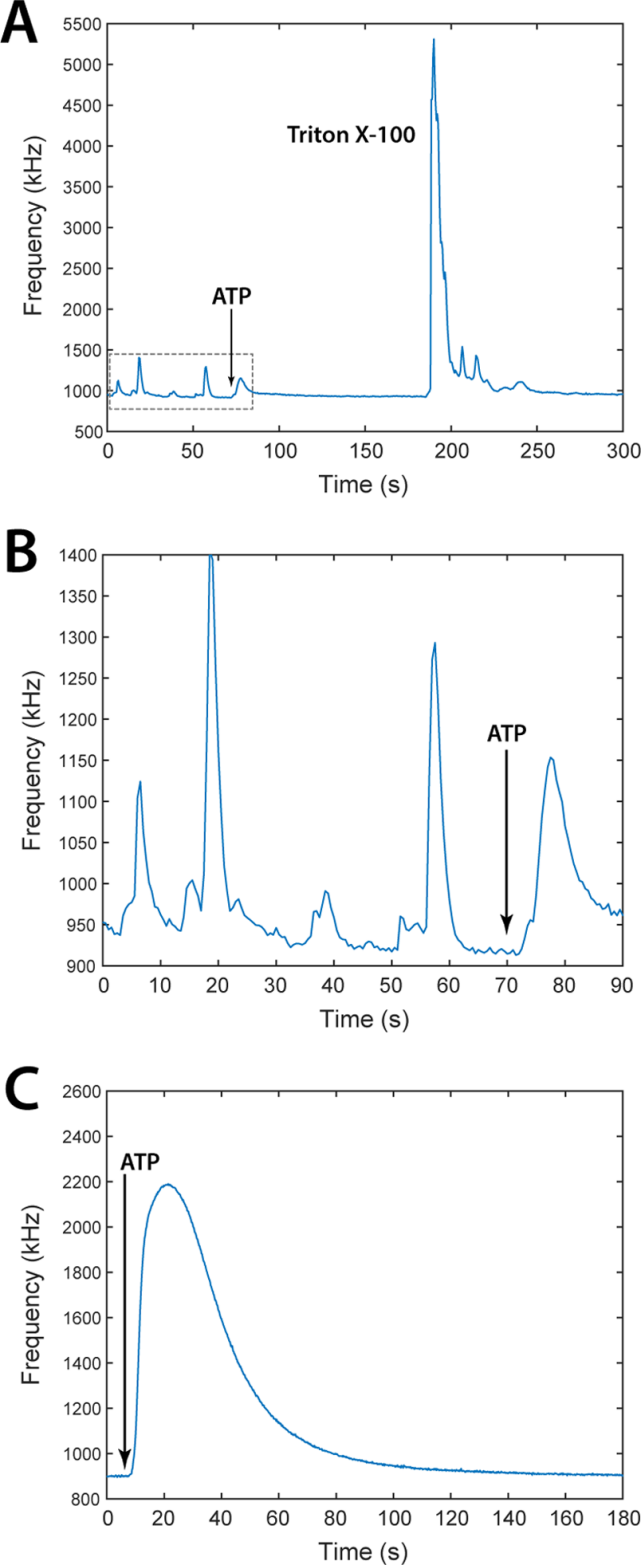
Bioluminescence signals from intact cyt- and mit-AEQ-transfected HeLa cells, recorded through PSAU discriminator in the photon-counting mode. (A) Representative frequency vs time trace corresponding to a plating density of 3000 cells/well. ATP (100 μM) and Triton X-100 signals are labeled in black. (B) Magnification of the gray dashed rectangle in (A) showing spontaneous calcium oscillations. The fact that these signals were completely abolished by ATP administration further confirms their biological, i.e., non-instrumental, origin. (C) Representative trace of ATP-induced [Ca^2+^]_m_ increases recorded from a cell culture sample at a plating density of 200,000 cells/well.

In order to explore the possibility of detecting extremely low-intensity signals, similar experiments were carried out in the SPC modality. Through this configuration, it has been possible to detect ATP-induced calcium signals in a wide range of plating density conditions, down to 700 cells/well. An exemplary trace is shown in Figure 7A. Occasionally, also tiny (less than 50 kHz) spontaneous [Ca^2+^]_c_ oscillations could be recorded prior to ATP administration (Figure 7B).

Spontaneous calcium oscillations are important and ubiquitous calcium-based signaling mechanisms. Like other calcium signals, oscillations depend on the cell expression of a sophisticated protein “toolkit” for membrane transport of calcium ion, such as ion channels, transporters, and pumps.^3^ Their generation is often the result of a coordinated interplay between a calcium release from intracellular stores and a calcium influx across the plasma membrane controlled by Ca^2+^ itself or by an increasing number of intracellular messengers.^3,26^ A large amount of studies indicate that spontaneous oscillations control a plethora of fundamental cell programs, such as fertilization, migration, differentiation, and maturation.^3,27^ In particular, in HeLa cells, spontaneous calcium oscillations with a behavior similar to the one showed in Figure 7B are thought to play a role in driving cells through the different stages of the cell-division cycle.^28^

One of the key features of AEQ-based Ca^2+^ probes is the ability to measure [Ca^2+^] in different subcellular locations.^16,29^ To prove that SiPM is also suitable to detect Ca^2+^ signals from an AEQ probe targeted to an intracellular organelle, we transfected HeLa cells with AEQ targeted to the mitochondrial matrix (mit-AEQ).^16^Figure 7C shows that the ATP-induced [Ca^2+^]_c_ increase induced Ca^2+^ uptake by mitochondria^30^ with a robust elevation of [Ca^2+^] in the mitochondrial matrix ([Ca^2+^]_m_). The dynamics of this elevation closely resembled that of mit-AEQ traces recorded using conventional aequorinometer setups.^16,29^ The trace peaked at 2200 kHz, which was far below the pile-up threshold (≈3100 kHz, Figure 3). In this experiment, a cell density of 200,000 cells/coverslip was used, indicating that the desired intensity of the signal can be easily obtained by downscaling the number of plated cells.

In general, as already stated with respect to linearity assessment, this front-end configuration featured the highest sensitivity (Figure 2), but it also proved to suffer from extensive pile-up, leading in turn to an event miscounting above 3 MHz (Figure 3). In particular, during the Ca^2+^ response elicited by Triton X-100, the system experienced extreme pile-up, while ATP-induced and spontaneous signals were correctly counted even at their peak amplitudes. As expected, the poor dynamic range of this configuration led to a significant increase in the *A*/*T* ratio (*A*/*T*_PSAU_ = 0.134 ± 0.015) with respect to the values obtained with both the aequorinometer and the CI-operated SiPM.

## Discussion

In this work, we addressed the challenge of using a SiPM sensor to detect the luminescence emitted upon Ca^2+^ release from living cells expressing aequorin targeted either to the cytosol or mitochondrial matrix. The main electronic issues connected to the fulfillment of the essential endowments required to satisfactorily reconstruct the Ca^2+^ transients temporal profile, namely, high dynamic range, extreme sensitivity, and sharp temporal resolution, were met by devising a parallel-readout scheme allowing the processing of the SiPM output signal according to SPC and CI acquisition modes simultaneously. Very recently, Santangelo and co-workers^31^ had already undertaken pivotal efforts toward exploitation of SiPM in luminescence assays. Namely, in ref (31), they tested the linearity and sensitivity performance of a SiPM-based luminometer with an integrated microfluidics system in measuring the luminescence originating from ATP-driven luciferin oxidation. However, although of great interest, the prototype described in ref (31) was essentially an advanced cuvette-like system. Conversely, here we demonstrated the ability of working on intact cells in an aequorinometer-like experimental configuration. On their own, luminescence traces acquired in the CI mode from AEQ-transfected HeLa cells stimulated with ATP, and subsequently disrupted by injection of Triton X-100, exhibited profiles comparable to those from a custom-designed PMT-based aequorinometer. Conversely, similar traces acquired in the SPC modality were found to be plagued by severe saturation of the Triton-induced luminescence burst due to massive pile-up effect, thus making CI acquisition the option of choice in luminescence experiments. Nevertheless, switching to the SPC mode proved to be mandatory in order to record even the weakest signals detected by the SiPM, such as spontaneous cytosolic calcium oscillations or ATP-stimulated releases from low-density cell cultures.

The SiPM-based system presented herein is not simply intended as a valuable and cost-effective alternative to currently exploited technologies (i.e., PMT-, EMCCD-, ICCD-, and sCMOS-based aequorinometers).^8^ Conversely, it claims to represent a strategic starting point toward the development of a new generation of luminometers that, by taking advantage of the peculiar features of SiPMs, will allow performing specific tasks, not necessarily restricted to calcium and aequorin domains, that are currently unfeasible through conventional PMTs. Here, it follows a non-exhaustive, albeit suggestive, list of such possible tasks.

SiPM matrices, which are currently commercially available, may be exploited for the design of multicolor luminescence detectors through the integration of proper mosaic filters over the sensor surface. Such a multicolor setup could be extremely appealing for the biophysicists and physiologists communities since it could enable the use of multitransfected cellular models. To this purpose, simultaneous transfection with cyt-AEQ and mit-AEQ could soon become the new standard for a more informative wide-spectrum [Ca^2+^] analysis. Alternatively, mit-AEQ and luciferase together could represent an unprecedented tool for studying the energetic balance of the cell, especially in those pathologies in which mitochondria and ATP metabolisms are known to play a fundamental role, such as Alzheimer’s disease,^32^ Parkinson’s disease,^33^ and heart failure.^34^ A multicolor system of this kind could be also suitable for applications relying on state-of-art bioluminescent ratiometric probes such as Lotus-V, a BRET-based indicator for transmembrane voltage measurements.^35^

Considering the typical size of a SiPM sensor and the compactness of both application-specific integrated circuits (ASICs) and dedicated front-end electronics, a rugged and portable setup for bioluminescence can also be conceived to be used with those particular samples, or in those particular environments, that require on-site measurements. Induced pluripotent stem cells (iPSCs), for instance, are a delicate cellular model that requires specific competencies and expertise to be managed and cannot be easily delivered to distant laboratories for bioluminescence testing. On the other side, Antarctica and space missions are two realistic examples of extreme environments whose effects on cell physiology could be efficiently studied by means of such a compact setup.

A further notable field of application is represented by the market of microplate readers, currently used as the standard luminometry technology for parallel drug screening, especially in the academic context of biomedical research (e.g., Perkin Elmer Victor X/Nivo, Promega GloMax, and Molecular Devices SpectraMax). All the abovementioned instruments feature a single bulky PMT in their sensor stage, thus forcing the screened microplate to mechanically slide over it in a sequential mode and making parallel time-course recording impossible. The physical and economic scalability of SiPMs could pave the way to the implementation of new readers featuring a SiPM matrix with an independent SiPM sensor under each well of the microplate. This would allow a parallel and high-throughput real-time acquisition for each biophysical or biochemical parameter for which a light-emitting probe is, or will be, available (e.g., membrane voltage, pH, [Ca^2+^], [ATP], [NAD(+)]/[NADH], and [AA]).

As a final note, with the advancement in SiPM technology, it is likely to soon witness a progressive improvement of the general features of these devices, such as shorter pulse durations (leading to higher performances especially in the SPC mode), smaller microcell pitches, lower DCR levels, etc. This will foster new applications such as bioluminescence imaging of single cells or even subcellular compartments.

## Conclusions

In this article, we provided a proof of principle for the time-course recording of intracellular Ca^2+^ dynamics using an aequorin-based probe and a SiPM sensor as the bioluminescence detector. In general, our experiments provide evidence of linearity of the overall system across three orders of magnitude of aequorin concentration. Charge-integration and photon-counting modes proved to be two alternative and complementary modalities for handling the SiPM output signal, coping with strong and weak bioluminescent signals, respectively.

The unquestionable benefits of solid-state electronics with respect to PMT technology, together with the rapidly improving SiPM performances, promise a bright future to bioluminescence measurements according to the methodology presented herein.

## Supporting Information Available

This file contains details about the pole-zero cancellation circuit used in the SPC branch, further exemplificative traces from the set of experiments performed on cell lysate comparing CI and SPC modalities at the two edges of the explored dynamic range (high and low AEQ concentrations, respectively), and a schematic description of the PMT-based aequorinometer used as reference setup (PDF)

## Footnotes

1 Considering the SiPM gain equal to 1.7 × 10^6^ and 1 ADC = 100 fC by datasheets.
