# Supplementary material for: Assessment of a Silicon-Photomultiplier-Based Platform for the Measurement of Intracellular Calcium Dynamics with Targeted Aequorin
Source: ACS Sens. 2020 Jul 23;5(8):2388–97. doi: 10.1021/acssensors.0c00277 (PMC8009593; doi:10.1021/acssensors.0c00277)
Supplement: Supplementary file 1 — se0c00277_si_001.pdf [file se0c00277_si_001.pdf]

Assessment of a silicon-photomultiplier based platform for the measurement of intracellular calcium dynamics with targeted aequorin

SUPPORTING INFORMATION

Federico Alessandro Ruffinatti[a,§], Samuela Lomazzi[b,§], Luca Nardo[b], Romualdo Santoro[b], Alexander Martemiyanov[b,c], Marianna Dionisi[a], Laura Tapella[a], Armando A Genazzani[a], Dmitry Lim[a], Carla Distasi[a,*], Massimo Caccia[b,*]

[a]Università del Piemonte Orientale, Department of Pharmaceutical Sciences, Via Bovio 6, 28100, Novara, Italy
[b]Università degli Studi dell'Insubria, Department of Science and High Technology, Via Valleggio 11, Como, Italy
[c]ITEP, Bol'shaya Cheremushkinskaya Ulitsa, 25, Moscow, Russia, 117218

[§]These authors equally contributed
*Corresponding author
Email: carla.distasi@uniupo.it, massimo.caccia@uninsubria.it.

Twin gain amplifier

The twin gain amplifier was designed as of the annexed schematics, where:

- The operational amplifier (AD8099) features ultra-low noise (0.95 nV/√Hz), and high gain bandwidth product (3.8 GHz), ideal for 16 bit systems and fast risetime signals;
- The card features two parallel output, 50 Ohm terminated, with gains at 34 dB and 20 dB, for low light pulses and for a high dynamic range;
- The output signal is characterized by a full time development of 150 ns, on both branches;
- For this specific application, the low gain branch was DC coupled, to cope with a high flux of photons in a severe pile-up situation.

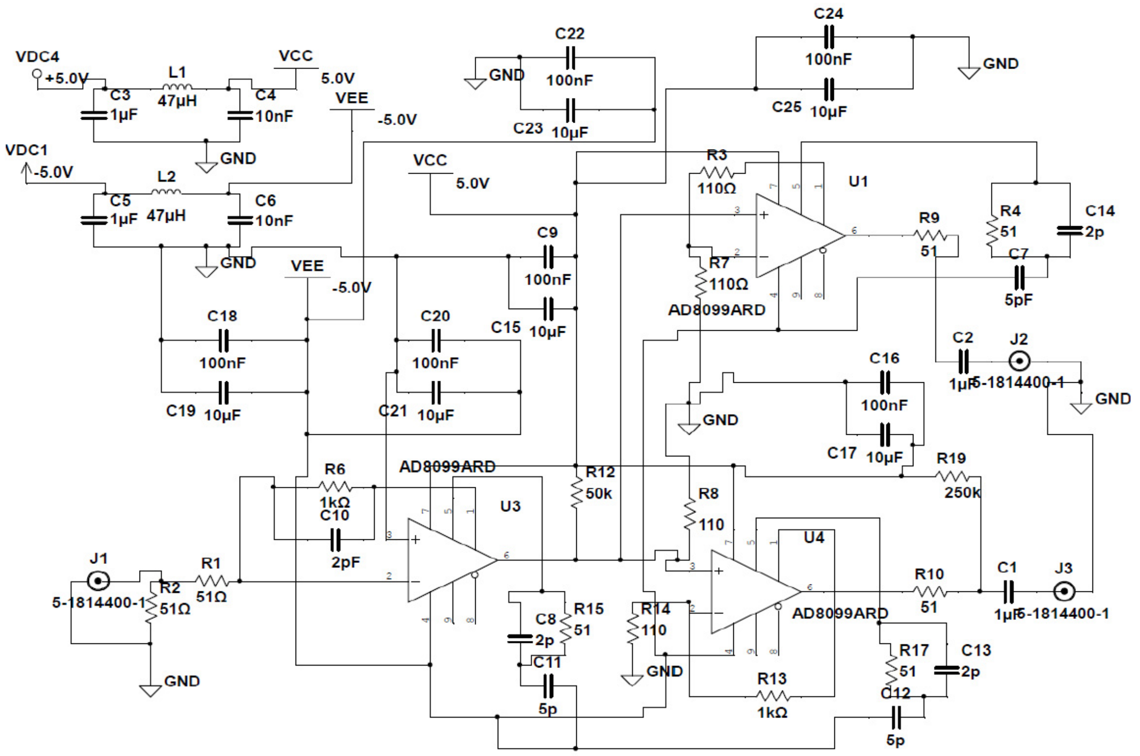

Figure S1. Schematics of the twin gain amplifier.

Pole-zero cancellation circuit

The pole-zero cancellation circuit is a rather classical passive circuit (see for instance Glenn Knoll, *Radiation Detectors and Measurements*, Third Edition, Wiley 2000, pp. 593-595) implemented according to this scheme:

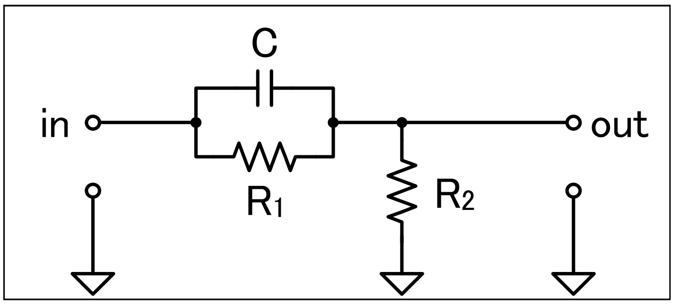

Figure S2. Scheme of the pole-zero cancellation circuit.

Presuming an exponentially decaying input signal with time constant τ_i, the components of the circuit are tuned to have $\tau_i = \mathrm{CR}_1$, so that the output signal is still exponential but with a shorter decay constant $\frac{1}{\tau_{\mathrm{output}}} = \frac{1}{\tau_i} + \frac{1}{\mathrm{CR}_2}$. The effect on the signal by the SiPM in use is evident in the following oscilloscope tracks

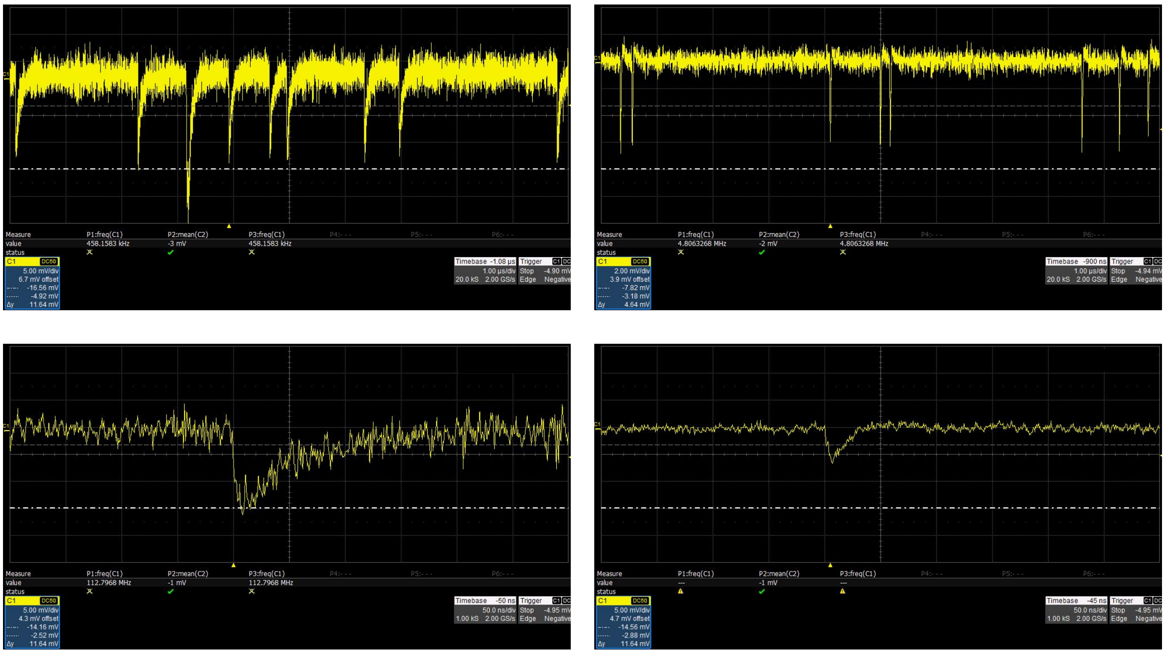

Figure S3. Full time development of the signal. *Upper oscilloscope screenshots show SiPM pulses in the absence (left side) and in the presence (right side) of the pole-zero cancellation circuit (time scale is 1 µs/div for both the traces). For both the conditions, a single-pulse magnification is shown in the lower oscilloscope screenshots (time scale is 50 ns/div for both the traces).*

where the full time development of the signal is reduced from 150 ns to 30 ns, with a clear advantage in term of pile-up reduction.

CI vs SPC for high AEQ concentrations

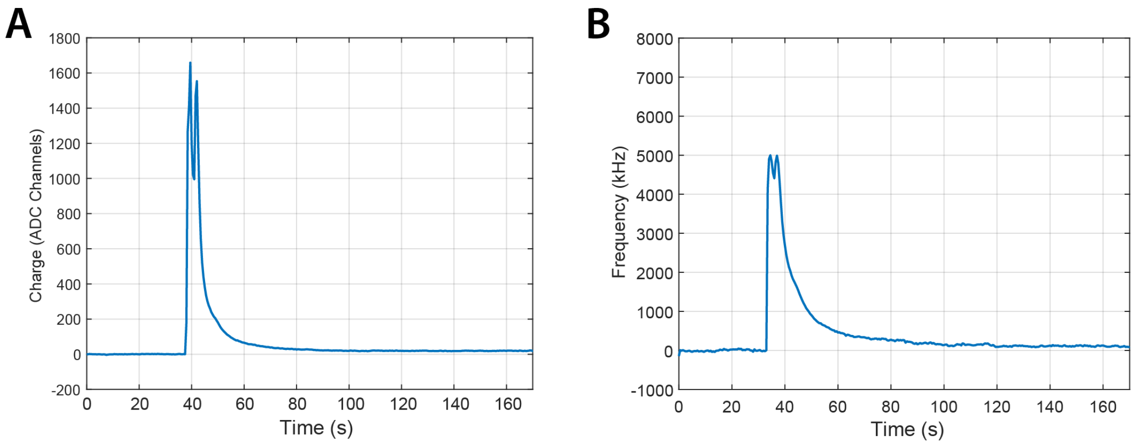

Figure S4: Advantage of CI over SPC modality for high AEQ concentrations. *Two representative traces recorded in CI* ***(A)*** *and SPC* ***(B)*** *modality for an [AEQ] dilution factor corresponding to* 2^{-2}*. By comparison, a slight deviation between the two traces is visible in the peak neighborhood because of the pile-up that affects the SPC mode.*

CI vs SPC for low AEQ concentrations

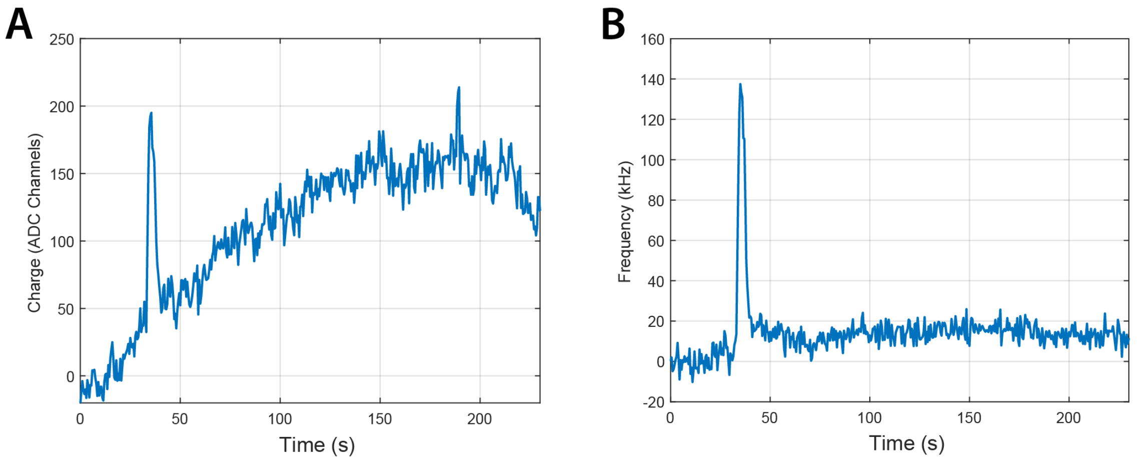

Figure S5: Advantage of SPC over CI modality for low AEQ concentrations. *Two representative traces recorded in CI* ***(A)*** *and SPC* ***(B)*** *modality for an [AEQ] dilution factor corresponding to* 2^{-9}*. A not faithful reproduction of the* Ca^{2+} *signal temporal profile in CI because of residual baseline fluctuations is shown.*

PMT-based aequorinometer by Cairn Research (UK)

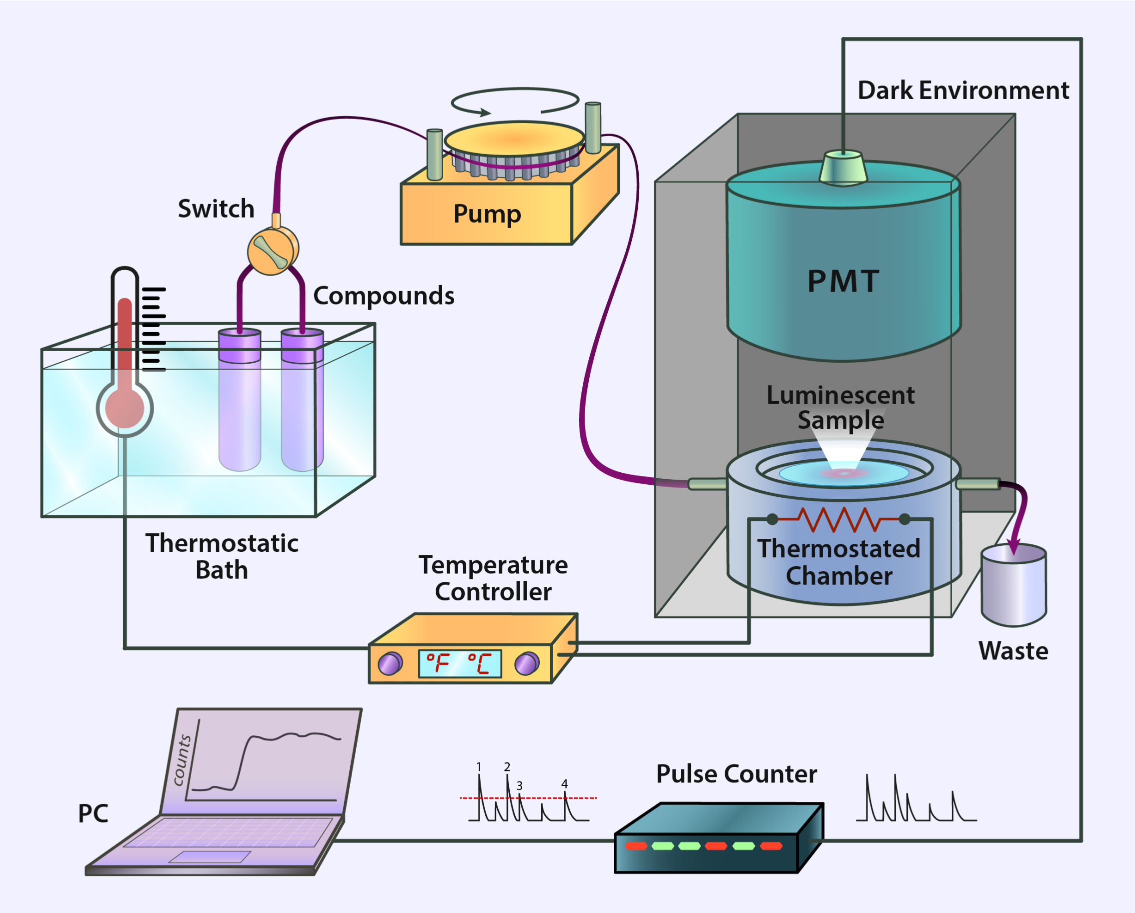

Figure S6: Scheme of the PMT-based aequorinometer used alongside with SiPM. To compare [Ca^{2+}] changes as recorded through our SiPM-based system with the known output of a well-established setup for aequorinometry, we used an aequorinometer built for purpose by Cairn Research (UK) with the following specifics. A steel thermostated chamber is used to host the coverslip with the adherent cells expressing the bioluminescent indicator of interest. The same chamber also features an inlet and an outlet allowing for a continuous perfusion of media and compounds under the action of a peristaltic pump. The temperature of both sample chamber and liquid media is set by means of a temperature controller. A photon-counting head (H7360-01 Hamamatsu Photonics) featuring a low-noise PMT is mounted over the thermostated chamber, in close proximity with the bioluminescent specimen. Both the photon counting head and the chamber hosting the cells are placed into a dark box to protect them from environmental light. Output pulses are fed to a photon counting board (Cairn Research), which is connected to a computer equipped with a dedicated acquisition software (Cairn Research).
